# A Rare Case of Meconium Periorchitis Diagnosed in Utero

**DOI:** 10.1155/2015/606134

**Published:** 2015-09-27

**Authors:** Daigo Ochiai, Sayu Omori, Toshiyuki Ikeda, Kazumi Yakubo, Tatsuro Fukuiya

**Affiliations:** ^1^Department of Obstetrics & Gynecology, Saitama City Hospital, 2460 Mimuro, Midori-ku, Saitama-shi, Saitama 336-8522, Japan; ^2^Department of Pediatrics, Saitama City Hospital, 2460 Mimuro, Midori-ku, Saitama-shi, Saitama 336-8522, Japan

## Abstract

Meconium periorchitis is a rare disorder caused by fetal meconium peritonitis, with subsequent passage of meconium into the scrotum via a patent processus vaginalis. To date, clinical significance of meconium periorchitis for the prenatal diagnosis of meconium peritonitis and prediction for postnatal surgery remains to be determined. We present a clinical course of a fetus presenting with meconium periorchitis induced by meconium peritonitis. At 28 weeks' gestation, fetal ultrasonography indicated fetal ascites associated with bilateral hydrocele and peritesticular calcification without other signs of meconium peritonitis. The pregnancy was uneventful until delivery and the infant was delivered at 37 weeks' gestation. No abdominal distension was observed at birth, and radiography did not reveal any abdominal calcification except for scrotal calcification. Abdominal distension was observed 3 days after birth and laparotomy was performed. The diagnosis of meconium peritonitis was confirmed at surgery. Our case illustrated that careful examination of the scrotum during fetal life was helpful for prenatal diagnosis of meconium peritonitis as well as postnatal management.

## 1. Introduction

Meconium periorchitis (MPO) is a rare disorder caused by fetal meconium peritonitis, with subsequent passage of meconium into the scrotum via a patent processus vaginalis [1]. To date, clinical significance of MPO for the prenatal diagnosis of meconium peritonitis and prediction for postnatal surgery remains to be determined. Herein, we present the clinical course of a fetus presenting with MPO induced by meconium peritonitis.

## 2. Case Report

**Figure 1:**
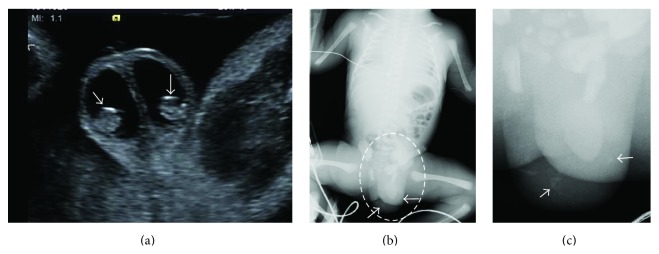
Prenatal ultrasonographic and postnatal radiographic findings. Prenatal ultrasonography revealed bilateral hydrocele with peritesticular calcification at 28 weeks' gestation (a), while postnatal radiography demonstrated scrotal calcification without abdominal calcification (b, c). (c) was an enlarged view of the dotted circle in (b). Arrow indicated calcification.

A 35-year-old gravida 1, para 1 woman was referred to our facility for evaluation at 28 weeks' gestation. Fetal ultrasonography indicated fetal ascites associated with bilateral hydrocele and peritesticular calcification (Figure 1(a)), leading to a diagnosis of MPO induced by meconium peritonitis, although other characteristic signs such as calcification in the abdomen, dilated bowel, and intra-abdominal cystic mass were not evident at that time. The fetal ascites disappeared within 1 week, and the bilateral hydrocele disappeared in 2 weeks. However, the peritesticular calcification persisted. At 30 weeks' gestation, a dilated bowel sign appeared, but it disappeared in 2 weeks. Viral infections including TORCH were negative. The baby was spontaneously delivered at 37 weeks' gestation, weighing 2545 g with Apgar scores of 8 and 9 at 1 min and 5 min, respectively.

No abdominal distension was observed at birth, and radiography did not reveal any abdominal calcification, but it revealed a scrotal calcification (Figure 1(b)), which was also demonstrated via ultrasonography. Abdominal distension was observed 3 days after birth; therefore, laparotomy was performed for the treatment of a bowel perforation. The diagnosis of meconium peritonitis was confirmed at surgery owing to the presence of a perforation of the small bowel 25 cm from the distal end of the ileum. The infant's scrotum remained asymptomatic and did not enlarge; therefore, the area was monitored carefully. The infant was subsequently discharged in good health.

## 3. Discussion

In the present case, fetal ascites was the isolated manifestation unaccompanied by other exudates such as cutaneous oedema, pericardial or pleural effusion, or polyhydramnios. Although the differential diagnosis of fetal ascites is diverse because of various possible causes including immune and nonimmune hydrops, isolated fetal ascites associated with scrotal hydrocele and peritesticular calcification are sufficient signs for the prenatal diagnosis of meconium peritonitis [2]. Prenatal characteristic findings of meconium peritonitis are abdominal calcification, ascites, dilated bowel, and intra-abdominal cystic mass [3, 4], but it is sometimes difficult to make a definite diagnosis because these signs vary in short periods. In fact, the fetal ascites disappeared within 1 week and no abdominal calcification was detected prenatally. In case of meconium peritonitis, Zerhouni et al. reported that fetal intra-abdominal calcification and dilated bowel on prenatal ultrasonograms predicted a need for postnatal surgery [4]. Since MPO is a complication derived from meconium peritonitis and not the other way around, careful examination of the scrotum during fetal life is helpful for the early and simple prenatal diagnosis of meconium peritonitis as well as its postnatal management.

A detailed evaluation of the scrotum during fetal life is significant to avoid an orchiectomy in the neonatal period. Jeanty et al. reported the radiologic findings of 48 patients with MPO diagnosed after birth [2], in which classical radiological findings in MPO, which are both abdominal and scrotal calcification, were detected only in 42% (20 patients). In the present case, scrotal calcification was prenatally detected, but abdominal calcification was not. Although MPO can be managed with either surgical or conservative treatment, most authors recommended that surgery be performed when a scrotal neoplasm is suspected [2, 5]. As MPO is known to spontaneously resolve, expectant management is acceptable. Therefore, careful examination of the scrotum starting in the fetal period is essential for noninvasive treatment of MPO in the neonatal period.

Thus, the present case highlights the clinical significance of a detailed evaluation of the scrotum in a case of isolated fetal ascites.
